# Twisted-Nematic Liquid Crystal-Infiltrated Bilayer Metasurface for Circular-Polarization LCoS Devices

**DOI:** 10.1021/acsaom.5c00640

**Published:** 2026-03-11

**Authors:** Xin Chang, He Ma, Mike Pivnenko, Weijie Wu, Yayan Tan, Jin Li, Daping Chu

**Affiliations:** † Centre for Photonic Devices and Sensors, 2152 University of Cambridge, 9 JJ Thomson Avenue, Cambridge CB3 0FA, United Kingdom; ‡ Cambridge University Nanjing Centre of Technology and Innovation, Site A 23 Rongyue Road, Jiangbei New Area, Nanjing 210000, China; § Institute of Information Photonics Technology, School of Physics and Optoelectronic Engineering, 12496 Beijing University of Technology, Beijing 100124, China; ∥ Engineering Research Center of Digital Imaging and Display, 12582 Soochow University, Suzhou 215006, China

**Keywords:** Circular dichroism, bilayer metasurface, spatial light modulator, liquid crystal on silicon, dynamic metasurface

## Abstract

Optical chirality underpins applications ranging from molecular identification to facial recognition. Metasurfaces have recently emerged as a versatile platform for compact chiral photonic devices. Here, we demonstrate electrically tunable circular dichroism (CD) at telecommunication wavelengths using a bilayer metasurface integrated with a twisted nematic liquid crystal (TN LC). The device comprises two silicon cuboid metasurface layers rotated by 30° relative to each other, with the interlayer gap filled by TN LC. The LC alignment was experimentally verified, confirming an effective metasurface-induced director orientation. Numerical simulations predict a maximum CD of 0.47 at 1575 nm, while experiments reveal an electrically tunable CD switching (ΔCD) of 8.2% at 1550 nm. This discrepancy is primarily attributed to lateral misalignment between the bilayer metasurfaces, as confirmed by numerical simulations. This architecture provides a practical route to extend conventional liquid crystal on silicon (LCoS) devices, typically designed for linearly polarized (LP) light, toward circular polarization-based LCoS (CP LCoS) devices, enabling opportunities for applications such as biomedical imaging and smart glasses.

## Graphical abstract

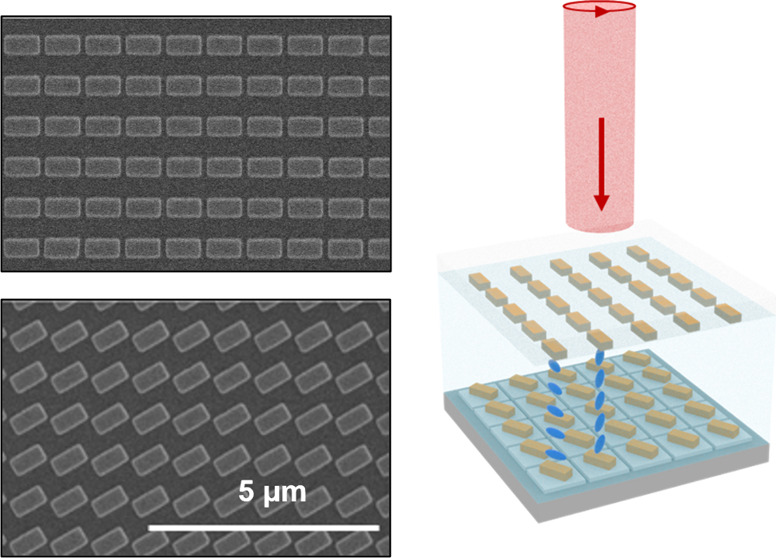

## Introduction

Geometric chirality arises from the breaking of the space parity symmetry. An intrinsically chiral object cannot be superimposed onto its mirror object (enantiomer) by any combination of rotation or translation operations, reflecting the absence of mirror symmetry in all spatial dimensions. With the rapid advancement of nanofabrication technologies, chiral metamaterials and metasurfaces with feature sizes comparable to the wavelength have emerged as a new paradigm for achieving strong optical chirality. Among these, metasurfaces have attracted particular attention owing to their compatibility with semiconductor manufacturing processes. A wide range of metasurface-enabled applications have been reported, including compact hybrid image projection system in augmented reality (AR) glass,^1^ photorealistic three-dimensional (3D) holography with a high space-bandwidth product (SBP),^2^ high-throughput structural coloration using low-index photoresist,^3^ and fast-switching telecommunication modulators.^4,5^

Chiral metasurfaces have been realized in both 3D and planar configurations.^6^ In 3D designs, chirality arises from broken out-of-plane symmetry. One approach employs metaatoms with height variations, where intrinsic chirality driven by bound states in the continuum (BIC) yields copolarized transmission differences up to 0.7 with quality factors near 80.^7,8^ Another strategy relies on slanted metasurfaces, where trapezoid nanoholes fabricated by angled etching allow control of the eigenpolarization at the Γ point by tuning both the in-plane trapezoid angle and the out-of-plane slant angle. When the circularly polarized eigenstate (C point) coincides with the Γ point in momentum space, BIC-driven resonant chirality with high CD is obtained.^9,10^ Bilayer metasurface represents another 3D strategy.^11^ For example, twisted silicon nanocuboids with C2 or C4 symmetry achieved CD values up to 0.7 through the formation of chiral supermodes,^12^ while elliptical^13^ or notched^14^ bilayer designs enabled both high CD (>0.9) and high-Q resonances by engineering near-field coupling and eigenpolarization control. At terahertz frequencies, bilayer photonic crystal slabs with combined in-plane and out-of-plane asymmetry achieved CD as high as 0.97 with Q factors exceeding 900.^15^ Tunable CD has been realized through several mechanisms such as the use of phase-change material,^16^ varying incident angle,^17^ mechanical stretching,^18^ and introducing electrostatic force.^19^ In addition to deliberately designed nanostructures, liquid crystals (LCs) have been widely employed to control chirality.^20^ Chiral liquid crystals (CLCs) are typically realized by doping nematic LCs with chiral additives, and they can also serve as soft templates for the self-assembly of chiral nanomaterials.^21^ More recently, the integration of LCs with metasurfaces has emerged as a powerful route to dynamic chirality control. For example, a nematic LC aligned at 45° relative to an elliptical silicon metasurface produced terahertz optical chirality, with electrically tunable co- and cross-polarized CD reaching 30 dB.^22^ A related bilayer design with an LC spacer enabled active control of symmetric and antisymmetric modes, yielding CD inversion over a regulation range from +28 dB to −32 dB at 0.47 THz.^23^ At near-infrared wavelengths, LC-integrated plasmonic metasurfaces have been proposed to achieve spin-selective absorption, including sign reversal of CD through LC reorientation.^24^ A dual-chiral platform combining a gold cilia metasurface with a chiral LC layer further demonstrated thermally reconfigurable CD, with experimentally tunable ranges spanning positive to negative values depending on LC thickness.^25^ These examples illustrate the versatility of LC–metasurface hybrids for reconfigurable chirality, although many reported devices remain limited to terahertz or plasmonic platforms.

In this work, we demonstrate an electrically tunable CD at telecommunication wavelengths using a bilayer metasurface with a 30° twist angle. The interlayer space is filled with nematic LC, providing reconfigurability, and together, the bilayer metasurface and TN LC constitute the overall device. The LC alignment on each metasurface was experimentally verified, confirming a relative director angle of 28°. Electrically tunable transmission for incident right- and left-circularly polarized (RCP and LCP) light was also measured, revealing a CD switching (ΔCD) of 8.2% at 1550 nm. This reduction in the experimental value is primarily due to lateral misalignment between the bilayer metasurfaces, as discussed in the final section.

The novelty of this work lies in the proof-of-concept demonstration of electrically tunable CD at telecommunication wavelengths by using LC-infiltrated bilayer metasurfaces. The proposed device introduces a new strategy for realizing electrically tunable chirality, enabling dynamic control of circularly polarized light in metasurface-integrated liquid crystal on silicon (MetaLCoS) platforms. This approach may further facilitate the development of circular-polarization-based LCoS (CP LCoS) devices. The approach promises applications including chiral biological imaging, augmented reality (AR), and virtual reality (VR) displays, advancing compact and electrically reconfigurable chiral photonic devices.

### Design and Simulation

The device comprises two stacked metasurface layers composed of silicon cuboids. Each unit cell has a period of 880 nm, with cuboids measuring 730 nm in length and 370 nm in width, as shown in Figure 1(a). The cuboids in the two layers are rotated by 30° relative to each other. The layers are separated by a 1500 nm cell gap, which is filled with nematic LC (extraordinary refractive index *n*_e_ = 1.8, and ordinary refractive index *n*_o_ = 1.5). The LC directors align with the orientation of the metaatoms, owing to the surface-induced alignment effect of the cuboid metasurface.^26^ As a result, the nematic LC adopts a TN configuration with a total twist angle of 30°. Numerical simulations were performed by using commercial full-wave software. Figure 1(b) shows the transmission spectra for LCP and RCP incident light (*T*_*L*_, *T*_*R*_) along the −Z direction, together with the calculated CD = *T*_*L*_ – *T*_*R*_ (black curve). A maximum CD of ∼0.47 is observed at a wavelength of 1575 nm, as indicated by the vertical gray line. For comparison, simulations were repeated for an identical cell without the metasurface layers, as shown by the dashed lines in Figure 1(b). In this case, both transmission spectra remain near unity, and the CD is negligible, confirming the essential role of the twisted bilayer metasurface in generating CD. The influence of LC reorientation on CD is presented in Figure 1(c). The dashed line marks the maximum CD at 1575 nm obtained in Figure 1(b). The LC rotation is quantified by the director angle θ relative to the XY plane. As the LC tilts toward the vertical direction (switch ‘on’), the CD at 1575 nm gradually decreases. This behavior is further elucidated in Figure 1(d), which plots the electric-field intensity distributions under opposite CP excitation. In the ‘off’ state (LC lying in the XY plane), RCP excitation yields stronger field enhancement in the bottom metaatom compared with LCP, giving rise to CD (see figures marked with black boundaries). In the ‘on’ state, however, the field distributions under RCP and LCP become comparable, leading to reduced CD.

**Figure 1:**
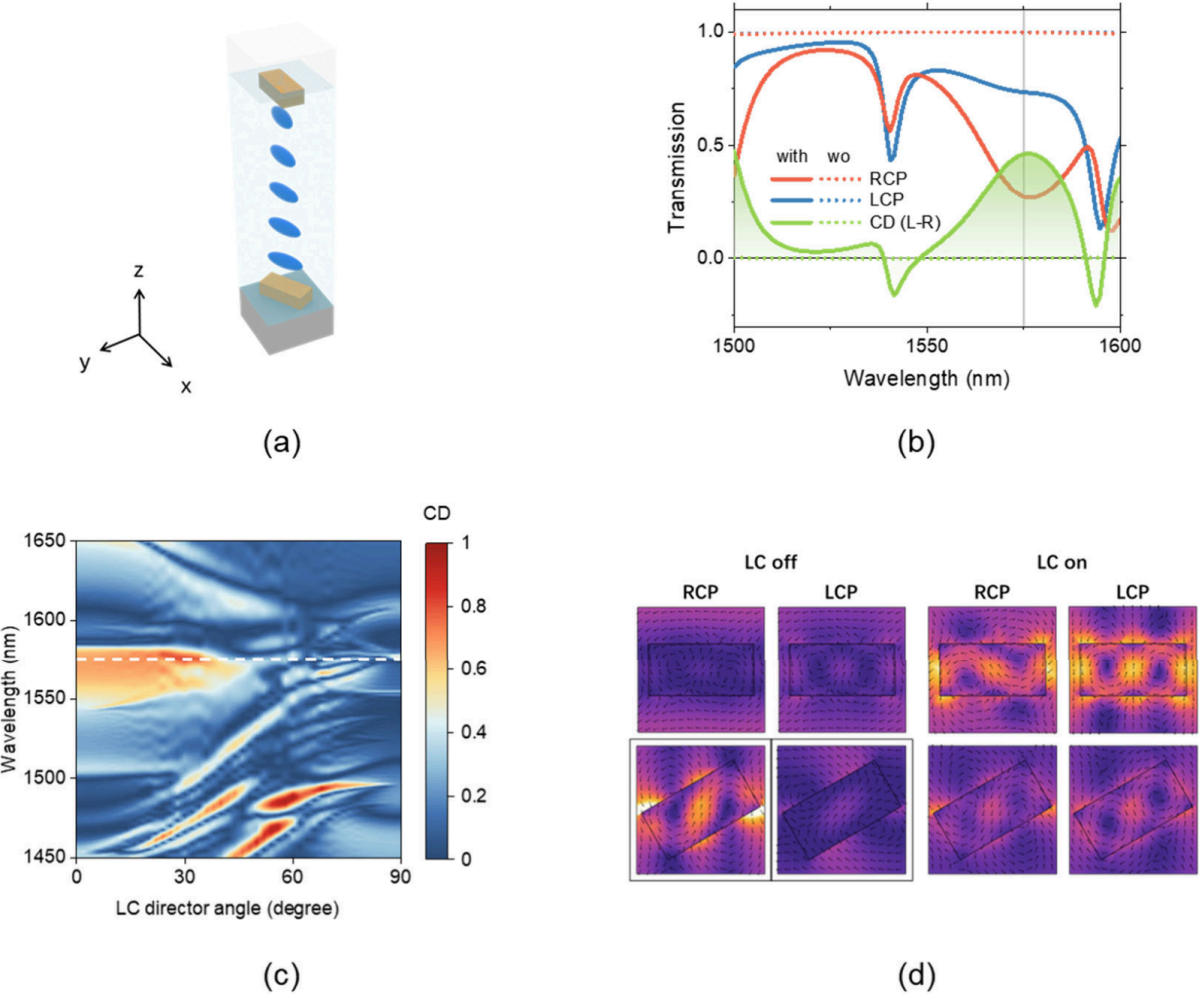
Numerical simulation of the proposed device. (a) Schematic illustration of the unit cell structure. (b) Transmission spectra and CD with (solid curve) and without (dotted curve) the metasurface. (c) Dependence of CD on the rotation angle of the LC. (d) Electric field intensity distributions under RCP and LCP incident light when the LC is in the ‘off’ and ‘on’ states.

## Experiments

The metasurface was fabricated by using a sequence of silicon sputtering, electron-beam lithography, liftoff, and reactive ion etching processes. The cell was then assembled with the metasurface encapsulated inside such that the two layers of the bilayer metasurface faced each other directly. The interlayer gap was defined by using 1.5 μm glass spacers. The chosen cell gap represents a compromise between achieving a strong chiral response and maintaining the fabrication feasibility. The cell gap was finally filled with nematic LC (GT7–29000, Merck) at 120 °C through capillary force. The assembled device was inspected under an optical microscope and mounted on a rotating stage between crossed polarizers. To examine the alignment effect induced by the metasurface, two reference (dummy) cells were fabricated, each containing a single metasurface layer with metaatoms oriented at 0° and 30°, respectively. Scanning electron microscope (SEM) images of the two metasurfaces are shown in Figure 2(a), along with optical microscope images of the corresponding dummy cells (M1 and M2) at in-plane rotation angles of α = 0° and 45°. Distinct optical transmission differences were observed between the two cells at identical rotation angles. The transmission as a function of α was further characterized using a photodiode (PDA36A2, Thorlabs), as shown in Figure 2(b). A pair of orthogonal linear polarizers was inserted into the optical path of the microscope. The device was mounted on a rotatable stage between the polarizers. Air was used as the reference for all of the measurements. For an ideal anisotropic medium placed between crossed polarizers, the transmission follows a sin^2^(α) dependence. Fitting the measured data with this model yielded a relative rotation of 28°, which is close to the designed value of 30°. The 30° rotation angle was chosen as it yields strong CD in simulations and provides an unambiguous alignment direction for the LC molecules. The small deviation is attributed to nonideal LC director alignment. As reported in ref 26, a minimum aspect ratio of 5 is required to achieve LC alignment quality comparable to that of one-dimensional gratings. The nanocuboids employed in this work have an aspect ratio of approximately 2, which is constrained by the geometry required to induce the desired electromagnetic response. Despite this reduced aspect ratio, reasonable LC alignment is still achieved, as made evident by the transmission contrast shown in Figure 2(c). An optical image of the assembled bilayer metasurface filled with LC is shown in Figure 2(c). The fabrication process of the metasurface (i–iv) and device (v–vii) was also illustrated. The measured optical transmission, plotted as square dots in Figure 2(d), is compared with the numerical simulation for a twisted-nematic LC layer with a rotation angle of 30° (solid curve). The good agreement between experiment and simulation confirms that the bilayer metasurface effectively induces the designed LC twist angle.

**Figure 2:**
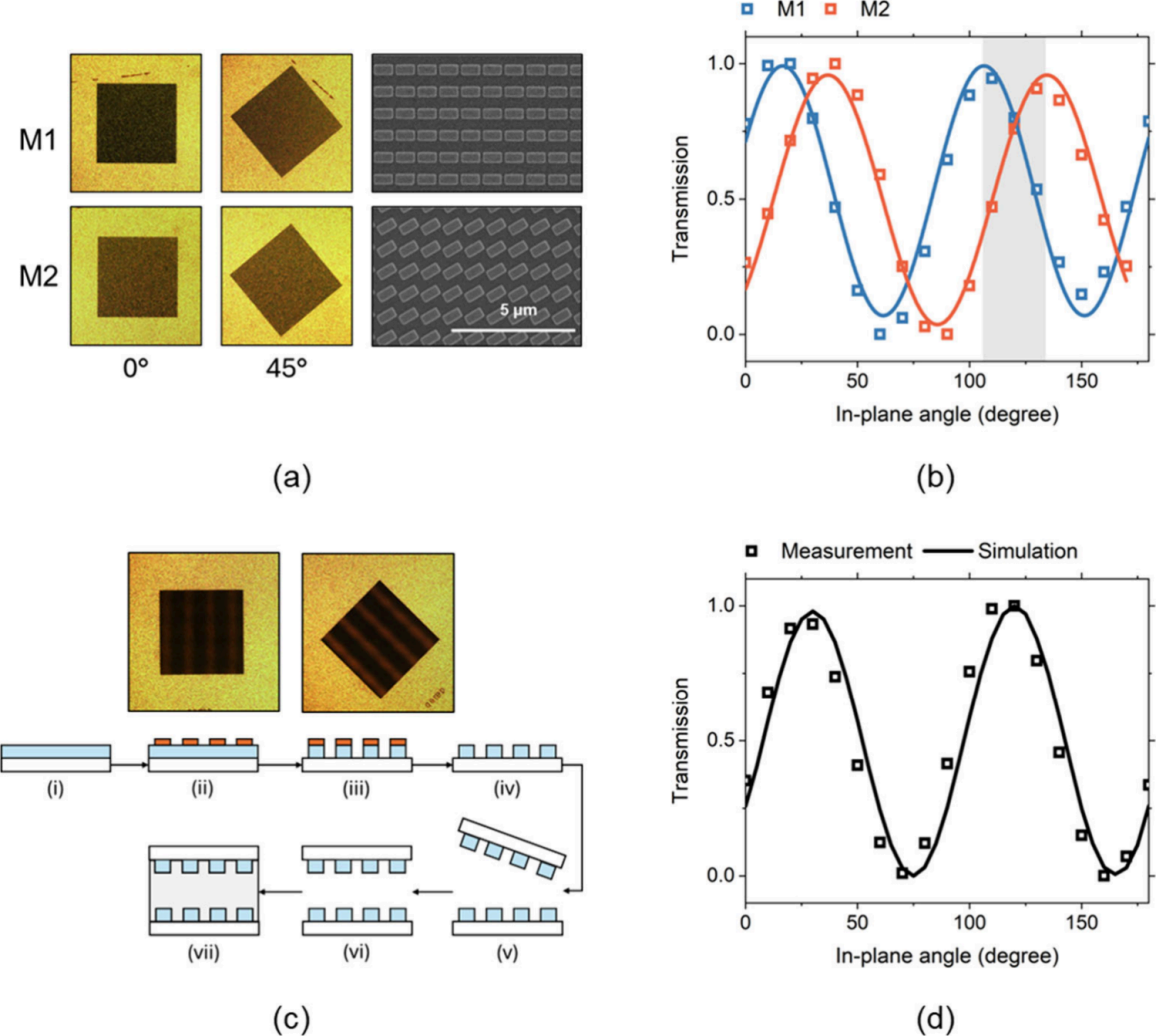
Experimental verification of LC alignment. (a) Optical microscope and SEM images of the fabricated metasurfaces. (b) Optical transmission as a function of in-plane rotation angle for the two metasurfaces. (c) Top: Optical image of the assembled bilayer metasurface cell filled with LC. Bottom: fabrication process. (d) Measured (square dots) and simulated (solid curve) transmission spectra of the bilayer metasurface cell.

The transmission spectrum of the assembled cell was measured under an applied AC voltage of 2 kHz. CP light was generated by placing a linear polarizer at 45° relative to a quarter-wave plate in the optical path before the sample. A second combination of a quarter-wave plate and a linear polarizer was positioned after the sample to selectively analyze the copolarized and cross-polarized transmitted light. Figure 3(a) and (b) presents the spectra for RCP and LCP incidence when the LC was in the ‘off’ and ‘on’ states, respectively. The peak driving voltage was 10 V. When the LC was in the ‘off’ state, the CD reached a maximum absolute value of 6.9% at 1577 nm. Upon switching the LC to the ‘on’ state, the CD nearly vanished, exhibiting an almost zero value within the wavelength range of 1564–1588 nm. The cross-polarized transmission components are also plotted in Figure 3(a) and (b) as shaded regions. Notably, the cross-polarized transmission reached a minimum when the CD disappeared. The portion of cross-polarized component is lower than some reported values in static metasurface^27^ and tunable metasurface.^25^ The main reason for the reduced polarization conversion and hence the CD value is due to the lateral misalignment and cell gap uniformity, as discussed in detail in the next section. Figure 3(c) shows the four transmission components, LL, LR, RR, and RL (the first letter denotes the incident polarization, and the second letter the transmitted polarization), under various peak driving voltages. The copolarized components were consistently stronger than the cross-polarized ones, confirming that the device’s CD is primarily intrinsic. Figure 3(d) further illustrates the variation of CD as a function of the driving voltage, clearly demonstrating electrically tunable CD behavior. The maximum CD modulation (ΔCD) of 8.2% was achieved at 1550 nm. It is also noted that the CD is negligible at the wavelength of 1580 nm when the LC is off, while CD reaches −6.5% once the LC is switched on. The abrupt change of CD between 0 and 2 V is due to the threshold-like reorientation behavior of the nematic LC when the voltage is above the Fréedericksz threshold. Above 2 V, the variation in CD limited, likely due to strong anchoring of the LC molecules by the metasurface structures. This effect may be alleviated by surface functionalization of the metasurface before LC filling.

**Figure 3:**
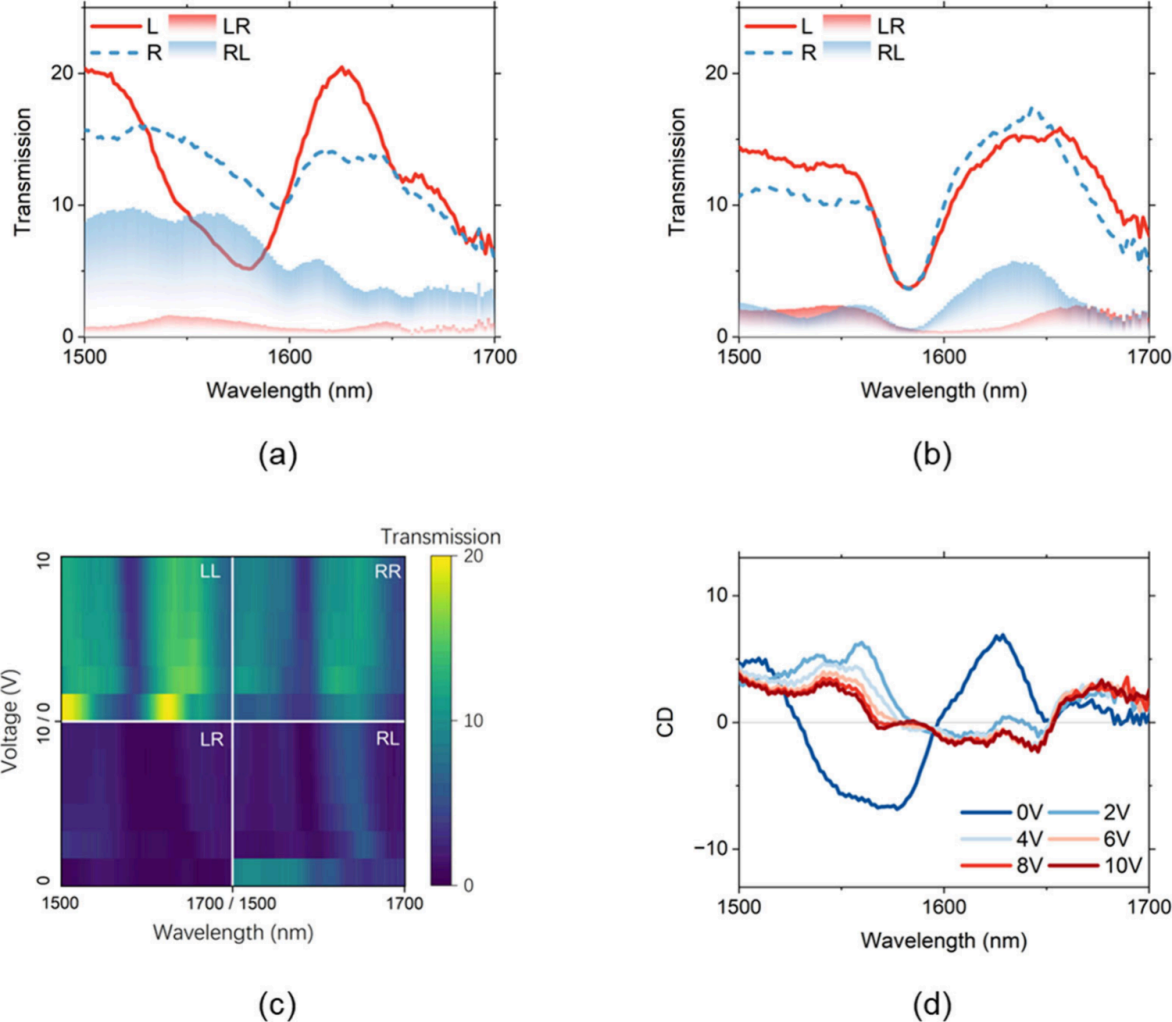
Experimental measurement of CD and its switching characteristics. (a, b) Transmission spectra for RCP and LCP incident light when the applied voltage is (a) off and (b) on. The shaded regions represent the cross-polarized components in the transmitted light. (c) Transmission of different polarization components (LL, LR, RR, and RL) under various driving voltages. (d) Electrically modulated CD as a function of voltage.

The experimental circular dichroism (CD) values obtained in this work are lower than those typically achieved with static metasurfaces without liquid crystal integration. For instance, CD values as high as 0.8 have been experimentally realized in the visible spectrum using planar gammadion metasurfaces.^28^ Nevertheless, employing finer nanostructures can enable both large CD and a wide CD tuning range. For example, nanocilia kirigami structures in conjunction with cholesteric LC have been used to experimentally achieve a CD tuning range of −0.76 to 0.84.^25^ In the terahertz regime, the elliptical silicon metasurface immersed in LC exhibited a pronounced CD of 30 dB.^22^ The following section discusses the origin of the weak chiral response and explores potential methods for its enhancement.

## Results and Discussion

In the simulation, perfect pixel-to-pixel alignment between the top and bottom substrates is assumed; however, achieving such precision experimentally is challenging without advanced alignment equipment such as a scanning electron microscope (SEM). The influence of lateral misalignment on CD is illustrated in Figure 4(a). When the liquid crystal (LC) is in the ‘off’ state, the CD variation (0.23) is smaller than that in the ‘on’ state (0.61). Moreover, cell thickness also has a nontrivial impact on the value of CD and its modulation range induced by LC switching (Δ*CD* = *CD*_*on*_ – *CD*_*off*_). The Δ*CD* range reaches 0.71 for a 1 μm cell gap but decreases to 0.19 when the gap increases to 3 μm, as shown in Figure 4(a) and 4(b). The reduction in Δ*CD* with increasing cell gap might be caused by the enhanced near-field coupling, which leads to the reduced phase retardation between LCP and RCP incident light and the reduced mode hybridization.^29,30^ These results indicate that both lateral alignment and cell gap uniformity strongly affect the absolute CD value and its modulation depth Δ*CD*.

**Figure 4:**
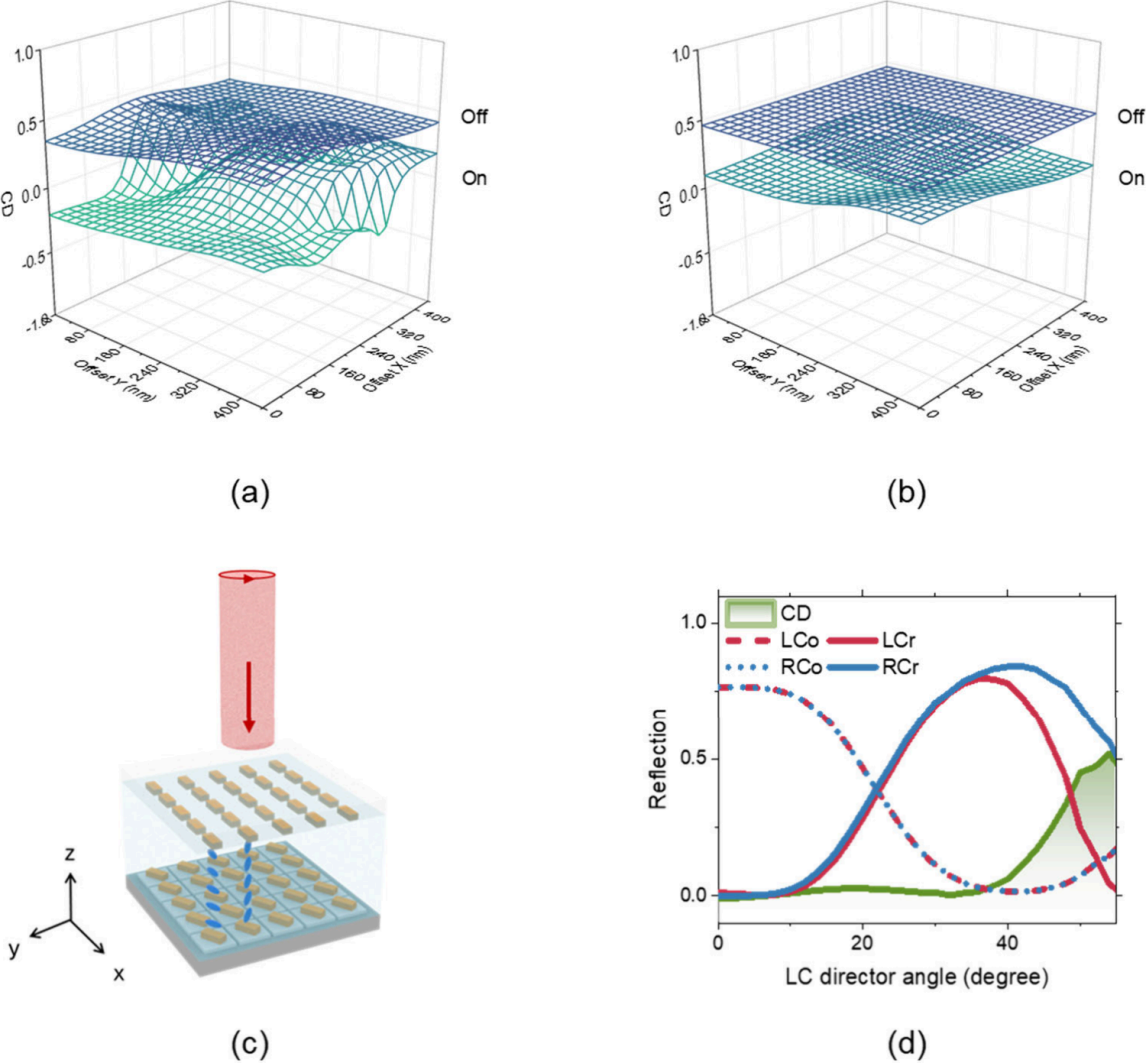
Numerical simulation of structural effects on CD modulation and the performance of the proposed CP-LCoS device. (a, b) Influence of lateral misalignment between the top and bottom metasurfaces on CD for the cell gap of (a) 1 and (b) 3 μm. (c) Illustration of CP-LCoS device. (d) Simulated reflection and CD at various voltages.

In this work, precise substrate alignment was not implemented, and therefore, some deviation between the measured and simulated spectra is expected. The modulation range could be further enhanced by improving the lateral alignment between the bilayer metasurfaces. Nevertheless, this proof-of-principle study successfully demonstrates a bilayer metasurface integrated with TN LC, enabling an electrically tunable CD.

Figure 4(c) schematically illustrates a CP-based LCoS device (CP LCoS). In contrast to conventional LCoS devices, which operate with LP incident light, the proposed CP LCoS is designed for CP incident light. Twisted bilayer metasurfaces are patterned on both the pixelated reflective backplane and the ITO-coated glass superstrate with a TN LC layer formed between them. The simulated reflection spectra are shown in Figure 4(d), where the applied voltage is represented by the liquid crystal director tilt angle with respect to the *z* axis. The co- and cross-polarized reflection components under LCP and RCP incidence are denoted as LCo, LCr, RCo, and RCr, respectively. When the LC is in the off state, the cross-polarized components (RCr and LCr) are strongly suppressed, in contrast to the handedness reversal typically induced by mirror reflection. The cross-polarized fraction is approximately 1% for the LCP incidence and below 1% for the RCP incidence. As the LC is progressively switched on, the cross-polarized components increase for both LCP and RCP excitation. At full switching, the copolarized fraction falls below 1% for both incident polarizations. In addition, the CD (defined here as difference in reflection between RCP and LCP: *R*_*R*_ – *R*_*L*_) with applied voltage and reaches a maximum value of approximately 0.52. These results validate the effective electrical modulation of CP light in the proposed CP LCoS device, highlighting its potential for CP-based light modulators (SLMs).

## Conclusion

In this work, we demonstrate a light-modulating device that can manipulate circularly polarized light at telecommunication wavelengths, as verified both numerically and experimentally. The device consists of two stacked bilayer metasurfaces with a layer of twisted nematic liquid crystals in between, achieving a maximum electrical switching in circular dichroism of 8.2% at 1550 nm. This architecture provides a practical approach to convert conventional LCoS devices, which typically operate with linearly polarized light, into devices that can modulate circular polarization. The proposed CP-based LCoS (CP LCoS) device is also numerically validated, exhibiting a simulated CD switching range from 0 to 52% as the liquid crystal is switched. The CP LCoS platform offers a compact and electrically tunable solution with potential applications in chiral biological imaging^31,32^ and augmented reality (AR)/virtual reality (VR) displays.^33,34^

## Data Availability

The data that support the findings of this study are available upon reasonable request.
